# Exploring recovery from anorexia in autistic adults: a qualitative study

**DOI:** 10.1136/bmjopen-2025-111034

**Published:** 2026-01-06

**Authors:** Abigail McIntosh, Rachael Hunter

**Affiliations:** 1 Swansea University, Swansea, UK; 2 Cardiff University, Cardiff, UK

**Keywords:** Eating disorders, QUALITATIVE RESEARCH, MENTAL HEALTH

## Abstract

**Abstract:**

**Objectives:**

To examine the barriers and facilitators of anorexia nervosa (AN) recovery in adults with autism.

**Design:**

Qualitative study using semi-structured interviews with autistic adults who identified as being in recovery or having recovered from AN.

**Setting:**

Participants were recruited via advertisements on social media and an eating disorder (ED) forum. Online Zoom interviews with 12 participants were conducted from October to November 2023.

**Participants:**

Overall, 12 autistic adults who identified as being in recovery or recovered from AN were included (11 women and 1 man; aged between 18–50 years).

**Results:**

Four key themes were identified: ‘Sensory Experiences’, ‘Recovery in progress’, ‘Changing to healthy mindsets’ and ‘Engaging with treatment’. Results indicated that recovery for participants did not follow a linear path, with the role of autistic traits, such as sensory sensitivities, interoception and the internal voice, making recovery challenging.

**Conclusion:**

This study provides insight into the challenges and motivations experienced during the recovery process. Findings highlight the need for further research to improve guidelines and autism awareness in ED services.

**STRENGTHS AND LIMITATIONS OF THIS STUDY**

- This study has provided a unique look into a group of autistic adults and aimed to enhance accessibility by including participants who were sensitive to social communication or face-to-face research.
- The interview guides were co-produced by academic researchers and autistic adults who were in recovery or recovered from anorexia.
- As recruitment occurred through volunteer sampling, there is a risk of sampling bias such as self-selection bias and thus there may be an inability to control for such biases in responses.
- Further research may benefit from a larger, more diverse autistic sample who are in recovery, or identify as being recovered from an eating disorder (ED), as evidence suggests that experiences can differ across different groups and EDs.

## Introduction

 Autism is a neurotype with diverse phenotypic variations that shapes the way individuals across the lifespan interact with and interpret the world around them.^1^,^3^ Autism is characterised by differences in socioemotional communication, repetitive hyperfocused patterns of behaviour and fixations on interests and activities, and a preference for routine, structure and predictability.^1^ These characteristics usually emerge in early childhood, but are not always recognised until later on in life.

The global prevalence of autism is 0.6%^4^ with a 3:1 male-to-female ratio for diagnosis.^5^ However, research suggests a sex and gendered diagnostic bias towards a conceptualisation of autistic traits predominantly found in boys and men. Autistic girls, women, transgender and non-binary people have heightened vulnerability to eating disorder (ED) development^6^,^8^ and may experience underdiagnosis or delayed diagnosis due to learnt coping strategies, including camouflaging (suppressing certain behaviours to appear neurotypical),^9 10^ which further hinders diagnosis.^2^ Additionally, autism is more likely to be misdiagnosed in racially marginalised groups, raising concerns over the diagnostic criterion used to assess autism.^8^ It is important to recognise that the prevalence of autism has increased over time, which is attributed to improved awareness, inclusion practices and changes in diagnostic criteria.^11^ However, gaps remain in understanding eating patterns in those with autism, given the higher vulnerability in this group to EDs.^7^

There is an estimated co-occurrence rate of 20%–35% between ED and autism,^12 13^ with 4%–52.5% over-representation of autism in anorexia nervosa (AN).^13^ Autistic individuals may experience significant delays in an AN diagnosis, with diagnostic delays of 14–22 years reported.^7 14^ Additionally, although AN is not the only ED co-occurring in autism, it is the most researched. Another ED that is commonly presented in both autism and AN, although not explicitly studied in this paper, is Avoidant Restrictive Food Intake Disorder.^15^

According to the fifth edition of the Diagnostic and Statistical Manual of Mental Disorders,^1^ AN is characterised by persistent restriction of energy intake that leads to significant weight loss, a preoccupation with food, an intense fear of gaining weight or behaviour that prevents weight gain (excessive exercise), restricted eating, bingeing and purging behaviours.

AN commonly develops in adolescence, with the highest incidence rates occurring from 15 to 18 years old.^16^ Although commonly diagnosed in girls (4% lifetime prevalence rate), AN also affects boys (0.3% lifetime prevalence rate).^16^ Additionally, individuals with AN may not seek or receive a formal diagnosis for their ED, which could lead to an underestimation of the true prevalence. Although 64% of adolescents with AN achieved full recovery after an average illness duration of 10 years,^17^ with relapse rates after treatment and weight restoration ranging from 30% to 72%.^18^ Meanwhile, 19% still met the criteria for an ED, and 38% received further mental health and physical diagnoses.

Common behavioural and socioemotional similarities have been identified between AN and autism, including sensory sensitivities, hyperfocused patterns of behaviour, communication difficulties and restrictive eating patterns (‘picky eating’), which can impact engagement with ED treatments.^13^ Hypersensitivity or hyposensitivity to taste, texture or smell can contribute to food aversion and restrictions, which are commonly reported in autistic adults.^19^ These hypersensitivities can lead to discomfort and distress, providing further motivation for restrictive eating^20^ and may be a factor in the continued vulnerability to AN and other EDs.

A study hypothesised that starvation associated with AN could strengthen cognitive rigidity identified in the AN population and be a central feature to the maintenance of AN.^21^ People with AN may have difficulty with implicit responses to emotional cues, including interpretation and responding,^22^ and have difficulty in explicit recognition of emotions.^23^ These difficulties are closely linked to impairments in interoception—the ability to perceive and interpret internal bodily signals, such as hunger and fullness.^24^ Rather than simply being a matter of not understanding hunger and fullness cues, recent evidence suggests that individuals with AN may have disrupted interoceptive awareness, which compromises their ability to identify and respond to internal states, potentially contributing to disordered eating. Importantly, similar interoceptive and emotional processing difficulties are widely reported in autistic individuals who often experience challenges in recognising and regulating emotions (as well as atypical interoceptive processing), which may further complicate their relationship with food.^25^ When AN co-occurs with autistic traits, these shared difficulties may exacerbate the severity and persistence of disordered eating behaviour, particularly when hunger is not accurately detected.^26 27^

A meta-analysis and review in autistic children reported problems in dietary intake and nutrition due to food selectivity,^28^ with feeding problems five times more likely than in neurotypical peers, often leading to inadequate dietary intake and nutritional imbalances. Indeed, a systematic review has reported ED symptoms in approximately 27% of autistic women,^19^ with a comorbid diagnosis of autism in around 5% of diagnosed ED cases. However, questions remain on whether these characteristics contribute to increased vulnerability to AN within autistic populations.^29^

Arguably, there is value in understanding the vulnerabilities to disordered eating for autistic individuals, as well as the role autistic features may play in recovery. Recovery from AN in autistic individuals presents unique challenges and often requires adaptation to standard treatment models. Not least because autistic individuals can find it more difficult to engage in ED treatment that is not designed for them.^26 30^ In a recently published study,^30^ it was reported that autism was considered a secondary issue by clinicians who were unclear on how to manage the co-occurrence, and autistic individuals often reported that their autistic traits were not adequately addressed, which reduced engagement and therapeutic efficiency.

Moreover, recovery in this population may look different from neurotypical pathways. For example, some autistic individuals may not identify with typical recovery narratives focused on body image or social eating and instead define recovery in terms of reduced distress, improved cognition and greater autonomy around food choices,^30 31^ potentially influencing reported recovery outcomes for these individuals.

There is a lack of in-depth exploratory study on subjective experiences and perspectives of recovery from AN in autistic populations.^32^ Existing studies often prioritise clinical outcomes, overlooking the personal and nuanced meanings of recovery for autistic individuals. It has been emphasised that there is an urgent need to explore how recovery is defined and conceptualised by autistic individuals themselves.^32^ Responding to this call, the current study aims to contribute to this underdeveloped area by adopting an exploratory approach that centres the lived experiences of autistic adults with AN.

This study seeks to explore how autistic adults with lived experiences of AN define and conceptualise recovery, what the perceived facilitators and barriers to recovery are within this population, and consider how sensory, emotional and social factors may influence recovery experiences.

## Methods

### Study design and setting

Autistic adults who self-identified as being in recovery or recovered from AN were invited to participate over Zoom from October to November 2023. The inclusion criteria were: (1) over the age of 18 years; (2) speak English fluently; (3) self-reported formal clinical diagnosis of autism; and (4) identified as being in recovery/recovered from AN.

### Participant recruitment

Participants were recruited through volunteer sampling, responding to the study advertisements posted on social media platforms (Reddit and Twitter), a national ED support charity (BEAT Eating Disorders Charity (https://www.beateatingdisorders.org.uk/), and the Eating Disorder Support Forum (https://www.edsupportforum.com/). No incentives were offered in exchange for taking part in the study. Data saturation was used to inform when to end recruitment.

### Data collection

Ethical approval was granted by the Swansea University Academic Departmental Committee (ref: 6207). All participants contacted the lead researcher to volunteer and were sent a participant information sheet, consent form and brief demographic questionnaire (eg, age, ethnicity and gender). All participants gave informed consent. Interviews were conducted from October to November 2023 in a private location ensuring confidentiality and conducted by the lead researcher over the Internet and applied inclusive approaches by using either video call (Zoom), with the choice to have the camera on or off, or Zoom chat. Semi-structured interviews were audio-recorded and lasted around 45 min (range: 26 min–1 hour 31 min) and consisted of nine open-ended questions (online supplemental file) exploring the experiences of participants, informed by relevant theory and literature.^33^ All participants were offered and received a debriefing session, and a debriefing sheet (with additional information for specialist support services) was sent to their email after the interview. Recordings were then transcribed, anonymised and participants were assigned pseudonyms.

### Data analysis

Verbatim transcripts of participants were analysed using the reflexive thematic analysis procedure.^34 35^ This technique was chosen because it is a flexible method to access a rich and detailed account of data and is a useful research tool for exploring the quality of life in health conditions and has been used in similar populations.^33^ After reading the verbatim data and listening to the recordings several times for accuracy and ‘data immersion’,^34 35^ initial thoughts and data extracts (‘quotes’) to identify patterns and themes related to the research question were manually coded. Relevant quotes were organised into meaningful themes with clear and concise definitions and placed into a visual table for better organisation. Extracts included in the agreed themes were discussed and reviewed by both researchers and checked back to raw data. To encourage reflexivity and reduce bias during the process, the lead researcher kept a reflective journal throughout the research process.^36^

### Patient and public involvement

Involvement of autistic adults with lived experiences of EDs was a central part of this research. The interview guide and research questions were informed by relevant theory and literature and explored what the facilitators and barriers to recovery were in this population. The questions were then presented on several ED sub-Reddits and the Eating Disorder Support Forum to help refine. All contributions were made by autistic adults with lived experience of an ED through the comment section of the post. After analysis of the data, potential themes were presented to the participants with a brief description of each theme to ensure a neuro-affirmative stance was used as neurotypical interpretations could be different from autistic interpretations.

## Results

A sample of 12 participants (11 women and 1 man) took part in the study. The decision to stop recruitment was informed by data saturation.^37^ Ages ranged from 18 to 50 years. Length of AN recovery ranged from 4 months to 12 years, and length of autism diagnosis ranged from 1 month to 40 years (table 1). Participants were asked to discuss why they considered themselves recovered and define what recovery looked like to them.

**Table 1:** Demographics for participants with autism and experience of AN (n=12*)*

Variables		(n)
Gender		
	Women	11
	Men	1
Age (years)		
	18–24	3
	25–30	6
	31–40	2
	50+	1
Country		
	USA	6
	UK	3
	Chile	1
	Norway	1
	France	1
Length of AN recovery		
	Under 1 year	1
	1–5 years	7
	6–9 years	2
	10+ years	2
Length of autism diagnosis		
	Under 1 year	5
	1–5 years	4
	10+ years	3

AN, anorexia nervosa.

Thematic analysis of interview transcripts identified four major themes and seven sub-themes that described the recovery experience of participants (table 2): ‘*Sensory Experiences*’*,* ‘*Recovery in progress*’*,* ‘*Changing to healthy mindsets*’ *and* ‘*Engaging with treatment*’.

**Table 2:** Main themes and sub-themes

Main theme	Sub-theme
Theme 1: Recovery in progress	Internal voice
Theme 2: Changing to healthy mindsets	Redefining eating
Theme 3: Sensory experiences	Disconnect from bodily cues
	Food aversions
	Need for control
Theme 4: Engaging with treatment	Motivations for treatment
	Experiences of treatment

These themes were closely linked, showing how sensory, emotional and systemic factors shaped the experiences of autistic adults with AN. Participants were anonymised by codes and supporting verbatim data extracts are presented for the themes.

### Recovery in progress

All participants emphasised that their recovery journey was “*not a linear thing*” (P5) and they have “*had slip-ups for sure*” (P3). To participants, recovery was an active process that required motivation and persistence, even when they did not feel like engaging:

I guess that is just part of recovery. I choose to eat and do the right thing even though my mind would still prefer to weigh 80 pounds (P1).

I struggle with that word ‘fully’ because I do think I am recovered … there’s this kind of ‘its definite’ and you’d never be vulnerable to that illness ever again which I think that’s kind of naïve thinking … like any mental health problem or like addiction it’s like an automatic thing to go to (P2).

Most of the time now I would say I am recovered. However, I do still have parts of me that are anorexic and still in anorexic thinking. Part of me wants to eat healthier and be healthy and be strong and muscular (P1).

Participants demonstrated a good awareness of what could cause them to “*slip*” (P2) or contribute to relapse— emphasising the dynamic nature of recovery.

It was a lot due to my parents commenting on my weight … it wasn’t just my parents, like there would be relatives stating if I gained weight, or if I lost weight and they made me feel conscious (P12)*.*

And although they may not be active within disordered eating behaviours, they are self-conscious about food and eating:

When I make food for me and my partner I’m always tempted to give them more than me … [If] I see that they’re eating, I’m always tempted to consciously eat less than them (P10).

Importantly, they went on to describe a range of coping strategies, including talking to friends and reducing stressors:

looking at content creators who focused more on body neutrality and body positivity, rather than looking at influencers who’s like ‘you can lose this much weight in 2 weeks (P12).

I am extremely privileged, and I have two sisters and several close friends who know I am … bonkers … and still show up (P4).

I had a really high stressed job and since being out of treatment, being in recovery, I’ve not been working in those types of roles … taking lower stakes work and managing how much work I have and how much I’m juggling is important (P11).

#### Internal voice

Most participants described a ‘voice’ in their head that reflected low self-worth, and that “*makes you convince yourself that you’re not worthy of anything or not deserving of anything*” (P5).

It kind of seeps its way through to different sort of elements of your life (P5).

[It] affected my self-esteem, and I would tell myself how stupid I was or how I did not deserve to live, or how I did not deserve to eat. So, I would not eat (P1).

For some participants, this inner voice had a strong influence and played a key role in enforcing rules and eating restrictions “*it’s very hard to break out of those … thoughts that led to the eating disorder … very hard to break rules*” (P6).

One participant experienced intrusive thoughts in their dreams, emphasising the consuming nature of the difficulties:

I often have nightmares too. Not only about weight, but about not being enough or the consequences of eating or people like dying and things … once I dreamt I died, and everyone was talking about how fat I looked at my funeral (P8).

### Changing to healthy mindsets

Participants described coming to a realisation that they “*don’t have to suffer anymore*” (P12) which helped them take control and challenge negative emotions and consciously make changes to enable a more autonomous and positive response “*in the name of recovery*” (P3). This was described by some participants as relearning or rewiring:

I’ve essentially had to rewire my thinking … I am not losing control by following a meal plan … I am making the conscious decision to eat … I am in control by saying that I will do these things (P3).

I learnt to not be so hard on myself … I learnt to eat more healthily. I learnt to keep exercise to under an hour a day, and I am learning how to get myself rest and a break after a busy day at work (P1).

Participants emphasised the practical benefits of fitness, consciously changing their mindset from a motivation to lose weight to a motivation to gain muscle and be able to engage in new lifestyle interests.

Weight doesn’t give you the full picture, it doesn’t give you your body fat percentage, it doesn’t give you like the strength that you have from certain physical activity (P12).

Noticing an influx of body positivity content on social media, some participants commented that beauty is not one-size-fits-all “*everybody’s healthiest looks different*” (P12) and this contributed to helping them adopt a proactive approach to managing triggers.

I try and actively avoid triggering content on social media, news articles, thinspiration stuff. That works most of the time (P5).

Social media and TV shows and movies … blatant fat phobia. And the way society makes it very hard to be okay with being in a larger body … there is no size that is healthier or attractive than another. And people in bigger bodies can be just as healthy, or healthier than people in smaller bodies, and just as beautiful (P6).

#### Redefining eating

Participants attributed having a support system as an important contributing factor to their recovery. The unconditional support from family members and friends was greatly appreciated and illustrated the profound difference that support can make in the recovery process:

I don’t think I’d be able to stay in recovery if I didn’t have my support system (P6).

My parents have been so supportive of me and my struggles with mental health as a whole in the past, I almost feel like I owe it to them to at least try … I missed out on so much when I was anorexic (P3).

Notably, the support system played an essential role in facilitating social eating. It enabled a safe, non-judgmental space for individuals to eat—for some, this alleviated pressure at mealtimes:

We set up a social club for watching movies, where we would always also have dinner and I’m aware that part of it was so I would eat dinner … just being with someone while eating, and where eating is something pleasurable, has been very invaluable (P4).

We are very far apart but we video chat with each other sometimes and eat dinner together, or she’ll send me recipes and help me learn to cook things (P6).

### Sensory experiences

Participants emphasised how traits (including sensitivities to smell, taste and touch) influenced their internal and external bodily perceptions and interactions with food groups.

#### Disconnect from bodily cues

This sub-theme reflected the hypersensitivity of participants to internal bodily sensations, leading to what was described as a sense of discomfort within themselves.

The way that my body works in terms of sensory… at all times I feel every single piece of clothing on my body, no part of it ever goes away (P10).

Participants described a disconnect from their body which resulted in difficulty interpreting internal bodily cues such as hunger. This was sometimes further complicated by emotions and the physiological effects of malnutrition, further reinforcing a complicated relationship with food:

I didn’t have a normal relationship with food … I don’t communicate very well with my body, so I don’t feel hungry necessarily and I don’t necessarily notice or make the connection that I’m being hypoglycaemic because I haven’t eaten (P4).

It made it difficult also to kind of understand how I was feeling a lot of the time, I knew I was upset about gaining weight, but I wasn’t upset necessarily, because I wanted to gain weight … It was just very confusing (P11).

Everything just felt wrong and that led to a lot of anxiety around my body and around existing in public spaces and a lot of depression from that disconnect from my brain, from what I was and what I should be. And the malnutrition probably added to that depression, because it’s very hard to get serotonin when you’re not nourished (P6).

#### Food aversions

Almost all participants experienced emotional and physical reactions when eating certain foods due to sensory sensitivities (ie, texture, taste and smell) and described it as “*torture*” (P7).

It’s also not disordered behaviour, it has nothing to do with me choosing to eat or not, it’s just a fact of life. It’s how I react to this experience (P7).

Aversion to certain textures, food combinations, temperatures and smells made eating a challenge and perpetuated avoidance and distress. One participant described the distressing experience of smelling a food they avoided as being like an “*extremely high-pitched squeal at all times*” (P7). For most participants, food restrictions were often associated with food-specific sensory sensitivities rather than the calorie or weight-related restriction typically associated with AN.

Certain smells and textures make me gag or make me sick to my stomach. It is frustrating because even when there are times that I want to eat or foods … if my senses can’t handle it, then I just can’t do it (P1).

I still would have very, very picky tendencies … not wanting anything to touch. Now, I’m not like that, I am so good, but I feel a lot more comfortable if I can just taste every component, then the whole thing all together (P7).

#### Need for control

All participants described restricting foods as a ‘habit’ that “*gives a sense of control, a sense of ownership, a sense of predictability*” (P5). Some participants named this as an interplay between autistic traits, sensory experiences, preference for routine and patterns, which exacerbated and perpetuated the ED:

There are some foods I hate because of the texture, but what I really do like is the routine. I do like knowing exactly what I’m going to eat, with who I’m going to eat with (P5).

With anorexia it wasn’t just food textures anymore, it was entire food groups … the level to which I became obsessed about the facts and figures of food, calorie labels … being autistic definitely made the depths of my obsession worse and the obsession was the main root of the anorexia (P3).

Mechanisms that maintained difficulties included self-imposed rules and restrictions driven by both the autism and ED thinking. This need for control and routine, and needing to have earned the right to eat, made recovery difficult, and one participant reflected on the central role of autism in the development of AN.

I’m also very rigid and categorical in terms of needing to have earned eating food (P4).

Who knows, without the autism, and by connection the obsessive nature that comes with it, maybe I wouldn’t have developed anorexia at all (P3).

### Engaging with treatment

#### Motivations for treatment

There were a variety of motivations for seeking treatment and focusing on recovery. For some, being able to have children was a main motivator, while others noted that they were “*just very sick of living*” with their ED (P11), feeling like they were dying or wanted to (“*suicidal ideation*”P10) and the cognitive and physical impact of AN also featured:

Not being able to remember things, or all of a sudden stuttering, or all of a sudden shaking to being out of breath … it scares me very much when I’m not able to access myself, like my brain, like I’m used to doing (P4).

I felt like I was dying … My body was shutting down, and my doctor said I had just a few more weeks to live if I did not get help. My heartbeat was slow, I had zero energy, I could not think (P1).

Nearly all participants remembered urgent referrals made by doctors or family members, while others sought help themselves. These acts of seeking or accessing support were defining moments in their recovery.

#### Experiences of treatment

Accessing ED services often took a long time, with some very unwell and in an “*acute state of illness*” (P2) before they were seen by healthcare specialists. Many described how autism did not seem to be considered when receiving treatment, and participants described often feeling misjudged by clinicians. In particular, autistic traits such as sensory experiences or preference for structure and detail were regarded as ED features and frequently led to participants being labelled as resistant to treatment.

Nobody really acknowledged my autism for a few months … and they were like ‘nah that’s just your eating disorder’ … I wasn’t given explanations for things which made me really distressed and just having panic attacks when it wasn’t about the food, it was about … nobody giving me clarity (P2).

Professionals could also be dismissive of their ED, stating “*you don’t look anorexic*” (P7). Regarding treatment, many felt “*there were parts of it that didn’t really land with me*” (P11) suggesting neurotypical language and approach. Overall, participants described that clinicians were “*not very well informed*” (P4) about autism, and this directly impacted on the therapeutic relationship and/or treatment engagement.

As treatment mainly focused on weight concerns, many patients described how their issues, which centred around interoception, were not fully examined or considered in assessment and treatment.

The treatment programme really focused on self-affirmation and self-worth and health at every size and body image stuff, you know, quitting social media and getting rid of bad influences … but it didn’t really help me deal with the sensory difficulties of … having my body changing rapidly or, you know, eating a lot of food, or dealing with digestion (P11).

They were very focused on whether or not I thought I was overweight, which I’m not delusional I know that I’m not, and I never had a specific like desire to look a certain way … I also think it’s pretty harmful to have that as such a big criteria (P4).

Despite these challenges, some aspects of treatment were beneficial. In particular, the physical environment of inpatient care, due to the “*containment and the structure of the* [*treatment*] *ward was definitely helpful and not being solely responsible for my own care*” (P2). In part, this was because it provided “*steps to follow and order*” (P6), thereby facilitating a pathway toward more independence and autonomy around food:

… you make your own meals so it’s kind of like a stepping stone … a test for me to see like if I could be responsible for myself and it gave me that chance to really like prove it to myself (P2).

## Discussion

The current study aimed to explore the lived experiences of AN recovery among autistic adults, with particular focus on how they conceptualise recovery, identify barriers and facilitators, and describe the influence of sensory, emotional and social factors on their recovery journey. Thematic analysis identified four major themes: ‘Sensory Experiences’, ‘Recovery in progress’, ‘Changing to healthy mindsets’ and ‘Engaging with treatment’.

Participants conceptualised recovery not as a fixed endpoint, but as an ongoing, non-linear process requiring daily commitment. None of the participants in this study were identified as ‘fully recovered’. They described recovery as ‘in progress’, acknowledging both setbacks and victories. This contrasts with conventional ED recovery models that often emphasise full symptom remission or weight restoration for recovery. However, this viewpoint is changing in the field as researchers realise that recovery definitions are not one-size-fits-all. There are competing perspectives on the meaning and definition of recovery.^38^ For these participants, recovery involved regaining autonomy, developing a healthier mindset, reducing anxiety around foods and improving sensory coping strategies.

Several unique facilitators and barriers emerged. Key facilitators included supportive, non-judgemental social relationships and a desire to regain autonomy. Strategies such as avoiding unhelpful social media content helped to shift mindsets, while social eating provided both practical tools and social support for navigating mealtimes. Although previous autism research has often highlighted reduced social relationships,^20 29^ in this study, social relationships enabled participants to feel accepted and not ‘othered’, positively supporting behaviour change and recovery. Many even adopted social eating as a strategy for maintaining healthy eating habits and reducing pressure around food scheduling, which contrasts with findings from ED research in non-autistic populations.^17^ This discrepancy may reflect that while social processing can affect eating in some autistic adults with AN,^26 27^ supportive environments can improve these interactions. Furthermore, research often overlooks differences in autistic and non-autistic communication styles, which may shape social interactions and influence how autistic adults experience recovery. More research in autistic lived experiences, particularly around social relationships, is essential for understanding recovery more accurately.^39^

In contrast, significant barriers included persistent sensory sensitivities, impaired interoception and the presence of an internal ‘anorexic’ voice. While the voice is widely acknowledged in ED treatment,^40^ participants highlighted that their difficulties with cognitive flexibility and rule-bound thinking made detaching from this voice harder.^21^ This suggests that the internal voice may operate differently in autistic individuals, potentially reinforcing fixation loops in ways that diverge from non-autistic experiences. Further research is warranted to understand how the anorexic voice functions within autistic cognition, including sensory sensitivities,^41^ and what this means for interventions.

Additionally, sensory and interoceptive experiences were foundational in both the onset and recovery process of AN. Unlike dominant ED theorists who emphasise body image disturbance as a primary causal factor (eg, the cognitive behavioural theory of AN^42^), participants described sensory overwhelm and confusion around hunger and fullness cues as central challenges, which contributed to anxiety around eating and avoidance behaviours—factors often underexplored in traditional models of ED. Emotionality and hyperfocused patterns of behaviour and fixations associated with autism intensified the difficulty of engaging in flexible eating behaviours and letting go of disordered rules. This aligns with previous research^25^ and supports the assertion that AN in autism may be less about body image and more about coping with overwhelming sensory and physiological experiences.^43 44^

This divergence from body image-focused narratives underscores a critical need to adapt treatment and conceptual models of AN for autistic populations. For example, while sensory sensitivities had been acknowledged in people with AN,^45^ its persistent and overwhelming nature in autism suggests it is not merely a symptom of starvation but a trait-like characteristic. Recovery approaches that fail to incorporate sensory sensitivities and interoception-related challenges miss core mechanisms sustaining disordered eating in this group.

In that vein, participants reported that their autism was often overlooked in treatment settings, with professionals focusing on standard ED protocols (eg, weight restoration) while neglecting the underlying autistic traits that contributed to the development and persistence of AN. Participants in this study also described a lack of understanding of autism and how it intersects with AN. These findings echo previous research showing that the standard ED treatment model often resulted in poorer outcomes for autistic adults unless modified,^3046^,^48^ and point to an urgent need for autism and AN training in autism for ED health professionals.

### Implications for further research

Individualised treatment approaches that integrate an understanding of the co-occurring diagnosis of autism and AN are essential. Ideally, there should be improved access to autism-specific ED services with, at a minimum, autism training for ED health professionals and, at best, regional specialists in autism and AN. Future research could survey the autistic experience and knowledge within specialist ED services. Similarly, future longitudinal studies into the presentation, experiences and outcomes for autistic people with AN are essential^32^ and may usefully contribute to the development of specific assessment tools and multidisciplinary models of care^13^ to enhance treatment adherence (coping strategies, relapse presentation and cognitive–behavioural techniques).^21^

A strength of this research is the use of a qualitative approach to better understand the lived psychosocial experiences of autistic adults who are recovering from AN.

However, this study is not without its limitations. Studies of this kind are at risk of participation bias, and it is unclear if these participants reflect a subgroup of individuals and as such, the experiences reported here should not be regarded as representative of all those with autism who are recovering from AN. To promote inclusion, this study recruited individuals who self-identified as being autistic with experience of anorexia, and as such, diagnosis is assumed but not medically confirmed.

This study was also comprised of mainly white, American women, which is a limitation, as demographic data on both sex and gender was not gathered. Therefore, it cannot be certain if participants identified as cisgender women or transgender women. Future research could explore the similarities and differences between demographics (eg, gender/sex, ethnicity). Finally, although all possible methods were used to ensure a good rapport between the interviewer and participant, some respondents could have felt uncomfortable revealing details of their experiences and/or experienced recall bias—not least because of reported cognitive decline during illness. Despite these limitations, the study provides valuable insights into the unique recovery experiences of autistic adults with AN.

## Conclusion

In summary, this study explored the lived experiences of autistic adults who self-identified as recovering/recovered from AN, identifying how recovery was navigated through evolving self-awareness, shifting mindsets and social support. The internal ‘anorexic’ voice, interoception, sensory experiences and lack of autism awareness in relation to AN were identified as barriers for effective treatment and recovery. These findings underscore the urgent need for more autism-informed, individualised approaches and AN training in autism for ED health professionals and services. Future research should expand to more diverse populations and explore how demographic factors (eg, gender/sex, ethnicity) and co-occurring conditions influence recovery to better inform inclusive and effective care.

## Supplementary material

10.1136/bmjopen-2025-111034 online supplemental file 1

## Data Availability

All data relevant to the study are included in the article or uploaded as supplementary information.
