# Supplementary material for: Exploring recovery from anorexia in autistic adults: a qualitative study
Source: BMJ Open. 2026 Jan 6;16(1):e111034. doi: 10.1136/bmjopen-2025-111034 (PMC12778344; doi:10.1136/bmjopen-2025-111034)
Supplement: online supplemental file 1 [file bmjopen-16-1-s001.docx]

**Interview Schedule**

Demographic questions:

*How old are you?*

*What is your ethnicity?*

*What gender do you identify with?*

*What country do you currently reside in?*

*How long have you been in recovery?*

*How long have you been diagnosed with autism?*

1. Introduction

*Can you tell me a bit about yourself and what motivated you to take part in this study?*

1. Experience of anorexia nervosa

*Can you tell me about your experience of anorexia?*

*Prompts*

*2.1. When did you notice your eating had become a problem?*

- - 1. *Did someone else point it out to you – friends/ family?*
    2. *Did you develop issues surrounding your body image or self-esteem? Tell me a bit about it.*
    3. *How did your experience with anorexia effect your mental health?*
    4. *How did it effect your physicality (activity level)? /Did you exercise for weight control?*

1. Causes and maintaining factors.

*Eating disorders can be difficult to understand, what other things do you think played a role?*

Prompts:

- 1. *Was your relationship with eating always difficult?*
  2. *What other things do you think played a role?*
  3. *Did you feel pressure to lose weight, or achieve a certain body type?*
  4. *What other things might have played a role in the development of anorexia?*
  5. *If they don’t mention ASC then could probe - and the ASC, how do you think this impacted – if at all?*

1. Eating disorder services

*Have you received support from eating disorder services or other health care services, and has that been helpful? Why/how?*

Prompts:

- 1. *Are you currently involved with them?*
  2. *Could you tell me about your experiences in these services?*
  3. *What was the referral process like?*
  4. *Who initiated your referral? How did you feel about being referred?*

1. Experiences of recovery

*Can you tell me a bit about what your recovery experience was/is like?*

Prompts:

- *What motivated you to recover?*
- *Are/were there any people who supported you (family, friends)? How did they support you?*
- *What has been helpful in your recovery process?*
- *What has been the most challenging?*
- *What would you say has been the things that has contributed to your recovery the most?*

1. Relationship between autism and eating disorder

*If you had to sum up your experience of AN, what role, if any, does/did autism play in your experience?*

Prompts:

- *Did you have sensory problems when it came to your eating?*

1. Summary

*Is there anything you would like to add? Any questions?*

*Thank you for your time. We’ll go through a quick debrief before we finish.*
